# On the Competition between Intergranular and Transgranular Failure within 7xxx Al Alloys with Tailored Microstructures

**DOI:** 10.3390/ma16103770

**Published:** 2023-05-16

**Authors:** Sutao Han, Matthieu B. Lezaack, Grzegorz Pyka, Nelson Netto, Aude Simar, Magd Abdel Wahab, Florent Hannard

**Affiliations:** 1 Institute of Mechanics, Materials and Civil Engineering, UCLouvain, 1348 Louvain-la-Neuve, Belgium; sutao.han@ugent.be (S.H.); matthieu.lezaack@uclouvain.be (M.B.L.); grzegorz.pyka@uclouvain.be (G.P.); nelson.gomes@uclouvain.be (N.N.); aude.simar@uclouvain.be (A.S.); 2 Soete Laboratory, Department of Electrical Energy, Metals, Mechanical Constructions and Systems, Faculty of Engineering and Architecture, Ghent University, B-9052 Gent, Belgium; magd.abdelwahab@ugent.be

**Keywords:** 7xxx Al alloys, ductility, formability, intergranular-transgranular competition

## Abstract

7xxx aluminium series reach exceptional strength compared to other industrial aluminium alloys. However, 7xxx aluminium series usually exhibit Precipitate-Free Zones (PFZs) along grain boundaries, which favour intergranular fracture and low ductility. In this study, the competition between intergranular and transgranular fracture is experimentally investigated in the 7075 Al alloy. This is of critical importance since it directly affects the formability and crashworthiness of thin Al sheets. Using Friction Stir Processing (FSP), microstructures with similar hardening precipitates and PFZs, but with very different grain structures and intermetallic (IM) particle size distribution, were generated and studied. Experimental results showed that the effect of microstructure on the failure mode was significantly different for tensile ductility compared to bending formability. While the tensile ductility was significantly improved for the microstructure with equiaxed grains and smaller IM particles (compared to elongated grains and larger particles), the opposite trend was observed in terms of formability.

## 1. Introduction

7xxx Al alloys are widely used in the aerospace industry, due to their high strength and fracture toughness. 7xxx Al alloys are heat-treatable alloys and can be strengthened through homogeneous precipitation of MgZn_2_ precipitates, up to a yield strength over 500 MPa in peak-aged (T6) condition [1,2]. However, strength and ductility are generally mutually exclusive and the lack of ductility of 7xxx Al alloys, when compared to other series of Al alloys, remains an important challenge for their applicability [3].

One of the main sources of damage in Al alloys is associated with micron-sized iron-rich intermetallic (IM) particles, as these particles may fracture or undergo interface decohesion [4]. However, in addition to these brittle particles, 7xxx Al alloys often exhibit grain boundaries, which are surrounded and weakened by a thin layer of material softer than the grain core promoting strain localization and intergranular fracture [5,6]. The competition between intergranular and intragranular mechanisms within 7xxx Al alloys is influenced by heat treatments and microstructural parameters [7]: (i) the quenching conditions are impacting the propensity to intergranular precipitation and grain boundaries embrittlement; (ii) aging heat treatments modify the state of the precipitates and precipitates shearing promotes localized shear bands and intergranular fracture; (iii) crystallographic and morphological textures are associated with preferential directions for intragranular crack propagation.

These softer zones, called Precipitate-Free Zones (PFZs), might be present due to two different phenomena [8,9]. The first phenomenon is associated with heterogeneous precipitation of coarse 𝜂 precipitates, which are trapping the alloying elements and generating a solute depletion in the vicinity of the Grain Boundary (GB). In this first case, PFZs are usually narrow bands with thicknesses smaller than 100 nm and contain a solid solution with relatively low solute content. The second phenomenon, usually observed for slow quenching rates, is due to the absence of a critical vacancy concentration in the vicinity of the GBs, which prevent the formation of hardening precipitates from the solid solution. In this second case, the PFZ thickness is of the order of a micron and contains a highly supersaturated solid solution. In both cases, the material inside the PFZ is very soft due to the absence of hardening precipitates. However, due to the very small thickness of the PFZ, the slip length available for dislocations is severely restricted, causing an initially steep work-hardening in the PFZ [10]. Furthermore, since the PFZ typically has a submicron thickness, strain gradient might affect its hardening capacity [11]. For PFZ which are much thinner than 1 μm, gradient effects on the hardening of the PFZ might be such that the PFZ becomes almost immediately harder than the grain itself [12]. The typical behavior of the material inside the PFZ will thus be a low yield stress and a high work hardening rate [9].

Friction stir processing (FSP) is a solid-state processing technology derived from Friction Stir Welding (FSW) [12,13], which can locally refine the microstructure by using a rotating tool penetrating into the material. FSP breaks the large iron-rich IM particles into smaller and more rounded fragments [14]. Furthermore, FSP also reduces the detrimental effect of IM clusters due to the local redistribution of particles. This refinement of the microstructure was shown to significantly improve the ductility in Al 6xxx [14]. In general, FSP leads to significant grain refinement in the Stir Zone (SZ), also called the nugget zone, due to dynamic recrystallization [15]. Due to the large deformation associated with FSP, deformation-induced substructures might result in a complex microstructure with different types of related boundaries, namely polygonized dislocation wall, partially transformed boundary and grain boundary [16]. This complex microstructure can have a determinant effect on mechanical properties and strengthening mechanisms in 7xxx series Al alloys [17]. Combined with a special cooling method, sub-micrometer grains have been observed in FSPed 7xxx Al alloys [18].

The stir zone is usually surrounded by deformed base material (BM) grains in the Thermo-Mechanically Affected Zones (TMAZs) and grains similar to BM in the heat-affected zones (HAZs). However, heat-sensitive precipitates of TMAZs and HAZs are influenced by the heat of the process, which might result locally in a lower hardness and promotes strain localization [13]. Post-FSP Heat Treatment (HT) is thus usually applied in order to ensure homogeneous hardness distribution. However, these treatments might lead to Abnormal Grains Growth (AGG) due to secondary recrystallization. AGG significantly deteriorates the mechanical properties and attention must be given to controlling the thermal cycles during processing in order to avoid this phenomenon [19]. Another approach to mitigate the heterogeneity of mechanical properties in the process zone is to use double-sided FSP, which was shown to produce a bimodal grain size in Al 7050, resulting in higher strength without significant loss of ductility [20].

The term formability can be misleading as it can be considered in terms of two limiting conditions: resistance to strain localization or resistance to ductile fracture [21]. Many sheet-forming operations are limited by strain localization, as typically characterized by a forming limit diagram. However, ductile fracture is usually the limiting condition in bending or under crash conditions [21,22]. Bending formability is of critical importance because bending is part of the deformation in many sheet-forming operations [23], such as door panel flanging or hemming processes [24]. In this work, formability will always refer to bending formability.

The objective of this paper is to investigate the use of FSP in order to produce 7xxx Al alloys with tailored microstructures and study their effects on the competition between transgranular and intergranular failure modes. 

Recent experimental results showed a 180% improvement of the fracture strain by combining FSP and post-processing HT on 12 mm thick Al 7475 sheets, without any decrease in the yield strength compared to the peak-aged (T6) base material [25]. In terms of bending formability, micro-cracks initiation on the outer tensile bend surface was delayed in the FSPed material compared to the base material. However, small FSPed grains (around 3 μm) favoured an intergranular failure mode, leading to fast crack propagation during bending [3]. These two opposite effects explained that the bending formability of the FSPed material was not improved compared to the base material, despite a significant improvement in terms of tensile ductility. 

In this work, FSP combined with post-processing HTs are applied on 2 mm thick Al 7075 sheets. By working on thin sheets, a refined FSP-T6 microstructure is produced in terms of IM particle size and initial porosities, but with grain size in the order of the base material (i.e., significant, but very homogeneous grain growth is observed within the stir zone). Indeed, the secondary recrystallization taking place during post-processing HT is highly sensitive to the cooling rate experienced by the material during processing [19]. This new microstructure is used to investigate the influence of the grain structure and IM particle distribution on the ductility and formability. These results are used to improve the understanding of the competition between intergranular and transgranular failure as a function of the loading conditions.

## 2. Materials and Experimental Methods

### 2.1. Materials and FSP 

**Figure 1:**
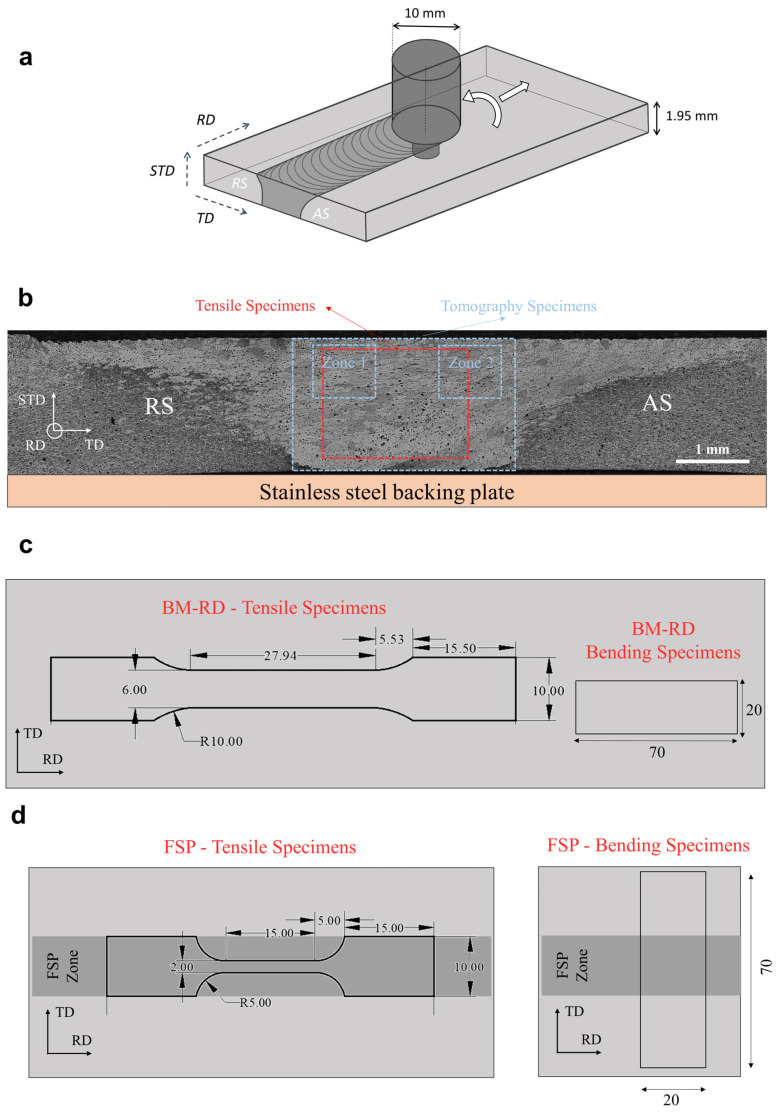
(**a**) Schematic of the FSP configuration and Al sheet (RD = rolling direction, TD = transverse direction, STD = short transverse direction) and (**b**) optical micrograph of the cross-section of the weld in a plane perpendicular to the FSP direction with visualization of the XRCT specimens, XRCT sub-specimens and tensile tests locations. Geometry and orientation of the tensile specimens and bending specimens in (**c**) the base material and (**d**) in the FSPed material.

Cold rolled 2 mm thin sheets of Al 7075 were used in T7 initial condition. Table 1 presents their chemical composition as measured by inductively coupled plasma-optical emission spectroscopy (ICP-OES). FSP was performed under displacement control with a tool rotation rate of 900 rpm, a traverse speed of 100 mm/min along the rolling direction and with a plunge depth of 1.9 mm. The FSP tool was composed of a 10 mm diameter shoulder prolonged by a 1.73 mm long tri-flat conical pin, 3 mm in reduced diameter and 3.2 mm in root diameter (Figure 1a).

A systematic decrease in the yield stress of 7xxx alloys has been shown after FSP due to coarsening of the hardening precipitates [3]. Post-processing HT was then applied in order to regenerate the hardening precipitates and recover a similar yield stress compared to the T6 BM [3]. First, a Solution Heat Treatment (SHT) of 30 min at 470 ± 5 °C was applied in an air furnace to FSPed samples followed by water quenching. Further artificial aging at 120 °C for 24 h was used to transform the FSP-SHT material into the hardest T6 state. 

### 2.2. SEM and X-ray Microtomography (XRCT)

Cross-sections for metallographic observations were taken along a plane perpendicular to the rolling or FSP direction. After standard polishing, microstructures were characterized using an optical microscope and a Field Emission Gun Scanning Electron Microscope (FEG-SEM) operated at 15 kV under secondary electron mode. When prepared for grain observation by optical microscopy, the samples were etched using Keller’s solution. 

As an increase of XRCT resolution usually comes at the expense of analyzed volume, XRCT was first performed with an isotropic voxel size of 1.44 μm, allowing to characterize IM particles and porosities over the full thickness of the sheets, and within a large part of the friction stir processed (FSPed) zone (Figure 1b). The XRCT at a voxel size of 1.44 μm will give access to the breaking and redistribution of large IM particles within SZ, but not to smaller particles and fragments. Sub-samples were then extracted in order to improve the resolution (voxel size of 0.6 μm). For the FSPed material, two sub-sample were extracted within the FSPed zone, both from the surface in contact with the FSP tool, with one sample on the advancing side of the FSPed zone (Zone 2, Figure 1b) and the other one on the retreating side of the FSPed zone (Zone 1, Figure 1b). Smaller and intermediate particles are thus characterized with the higher resolution scan (0.6 μm).

Intermetallic particles and cavities were segmented by manual thresholding. Labelling and parameter measurements were then performed using a dedicated image processing plugin implemented in ImageJ [26].

### 2.3. Microhardness

Vickers microhardness tests were performed using an EMCO-TEST Dura Scan G5. The test load was 0.3 kg (HV0.3) and the dwell time was 10 s. All samples were previously polished with a diamond paste with a particle size of 1 μm. A 5 by 60 indentation array was performed and centred at mid-thickness. Each line is parallel to the plate surfaces and comprised of 60 points spaced by 500 μm. Five lines are separated by 250 μm in the thickness direction. 

### 2.4. Uniaxial Tensile Tests

Tensile tests were performed using a universal Zwick 50 kN testing machine under displacement control, at a constant velocity of 1 mm/min. Flat dog-bone samples with two sizes were tested in the BM-T6 and in the FSP-T6 material. These dog-bone specimens, with a cross-section of 1.5 mm × 6 mm, were extracted from the BM-T6 (Figure 1c). The initial gauge length was equal to 22 mm. Smaller specimens, with a cross-section of 1.5 mm × 2 mm, were extracted in the processed material along the FSP direction. The initial gauge length was equal to 10 mm. This smaller geometry ensures that the tensile specimens are completely extracted within the process zone (Figure 1d). All samples were machined by wire-cut Electrical Discharge Machining (EDM), extracted at mid-thickness for base material sheets and centered in SZ for FSPed material (see Figure 1b). All tensile samples were polished with 1200-grit SiC paper. Three tensile samples were tested under each condition.

The initial yield stress *σ*_0_ is extracted from the stress-strain curves. The true fracture strain is defined as *ε_f_* = ln(*A*_0_/*A_f_*), where *A*_0_ and *A_f_* are the initial and final cross-section area, respectively. The final cross-section area *A_f_* was measured post-mortem on the broken specimens by optical microscopy. The fracture strain, *ε_f_*, corresponds to an average value of the true strain in the minimal cross-section of the specimen at fracture. The true fracture stress (defined as *σ_f_* = *F_f_*/*Af*) was computed from the last force value, *F_f_*, recorded before fracture and from the fractured section area, *A_f_*.

### 2.5. Plate Bending Tests

Bending coupons of 70 mm length and 20 mm width were extracted out of the BM-T6 and FSP-T6 materials, see Figure 1c,d. All the samples were extracted at mid-thickness and both sides were uniformly polished down to a thickness of 1.4 ± 0.5 mm. Samples were extracted in the BM with the bending line perpendicular to the rolling direction, therefore, producing tensile stresses on the outer surface along the rolling direction. FSP-T6 bending samples were extracted with the bending line centered and perpendicular to the processing direction (see Figure 1d), ensuring the bending of the specimen within the SZ. Three bending samples were tested under each condition.

Plate bending tests were performed following the standard VDA-238-100-2017 [27] and the bending setup is shown in the inset of Figure 8. The shut-off criterion was a 10% drop in force after the maximum force has been reached. The bending angle has been calculated from the stroke of the bending punch following standard VDA-238-100-2017.

## 3. Results 

### 3.1. Microstructure 

#### 3.1.1. Grain Morphology and Size 

**Figure 2:**
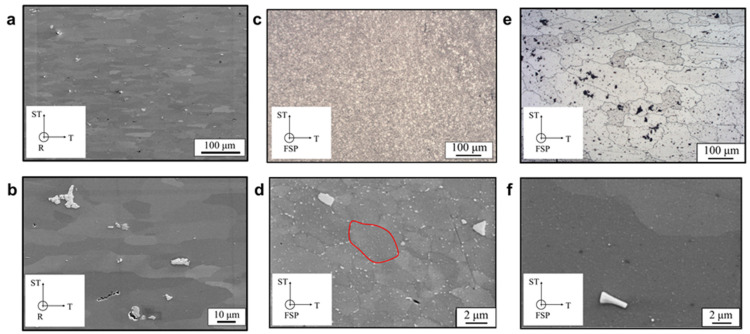
(**a**,**c**,**e**) Low and (**b**,**d**,**f**) high magnification micrographs of grain structures in the BM-T6 (**a**,**b**), as-FSPed (**c**,**d**) and FSP-T6 (**e**,**f**) materials, respectively. (**c**–**e**) are observed by optical microscopy after etching of the samples. (**a**–**e**) are observed by FEG-SEM. RD = rolling direction, TD = transverse direction, STD = short transverse direction. Please note the scale difference between (**b**) and (**d**–**f**). One grain has been drawn in red in (**d**) for easier comparison.

Figure 2 shows the grain structures of BM-T6, as-FSPed material and FSP-T6 materials. The base material (Figure 2a,b) is composed of elongated grains, with a size of around 100 × 10 μm in T-ST cross-section. Iron-rich particles, typically around a few μm, are distributed within the matrix.

Figure 2c,d shows the grain structures within as-FSPed material. After FSP, the shape and size of the grains have been significantly modified. The rolled grains were broken and recrystallized during processing due to the heat and mechanical stirring. The grains are almost equiaxed with a size of around 4.3 μm. Figure 2d shows many coarse precipitates that are distributed along the grain boundaries.

Figure 2e,f shows that the FSPed grains have significantly grown during the post-FSP T6 heat treatment, with a grain size of around 148 μm. The small grains generated during FSP have experienced secondary recrystallization during the T6 post-processing HT. It is interesting to note that this significant grain growth is quite different compared with previous results. Indeed, a similar procedure—FSP + T6 post-processing HT—performed on 12 mm thick 7475 Al alloy [19] lead to a final grain size of around 3 μm. In order to avoid abnormal grain growth during post-FSP heat treatments, it is necessary to reduce FSP temperature gradients through the plate thickness [19]. Grain growth by secondary recrystallization was thus favoured for thin sheets, but Abnormal Grain Growth (AGG) was not observed (i.e., significant, but very homogeneous grain growth is observed within SZ). Indeed, samples with AGG exhibit early tensile failure with a significant reduction of the true fracture strain [19], which is not the case for the FSP-T6 specimens of this work. This will be discussed in Section 3.2.2. Finally, coarse precipitates formed along grain boundaries during FSP (Figure 2d) have been dissolved during the post-process HT (Figure 2f) and a homogeneous distribution of small precipitates may be distinguished within the grains. 

#### 3.1.2. Intermetallic Particles and Porosities

**Figure 3:**
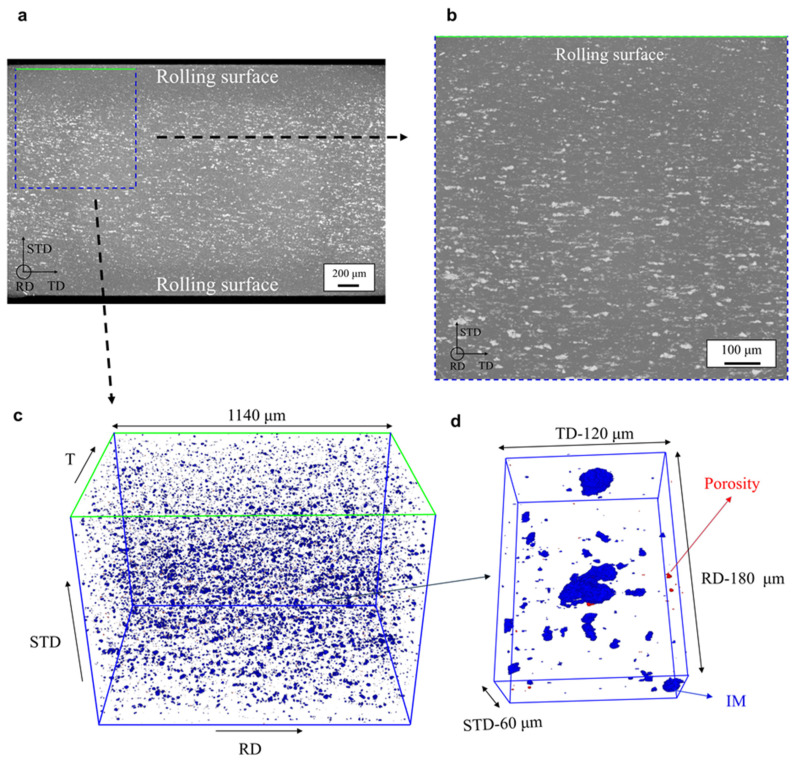
(**a**) 2D visualizations of IM particles created by taking the maximum intensity projection (MIP) of the lower resolution (voxel size of 1.44 μm) XRCT images of the BM over a 2 mm thick slab. (**b**) Similar 2D visualization for the higher resolution (voxel size of 0.6 μm) XRCT image of the BM sub-specimen over a 0.8 mm thick slab. (**c**) 3D XRCT perspective of particles (in blue) and cavities (in red) in the higher resolution XRCT image of the BM sub-specimen. (**d**) Zoom of (**c**), showing a few IM particles and an initial porosity. RD = rolling direction, TD = transverse direction, STD = short transverse direction.

**Figure 4:**
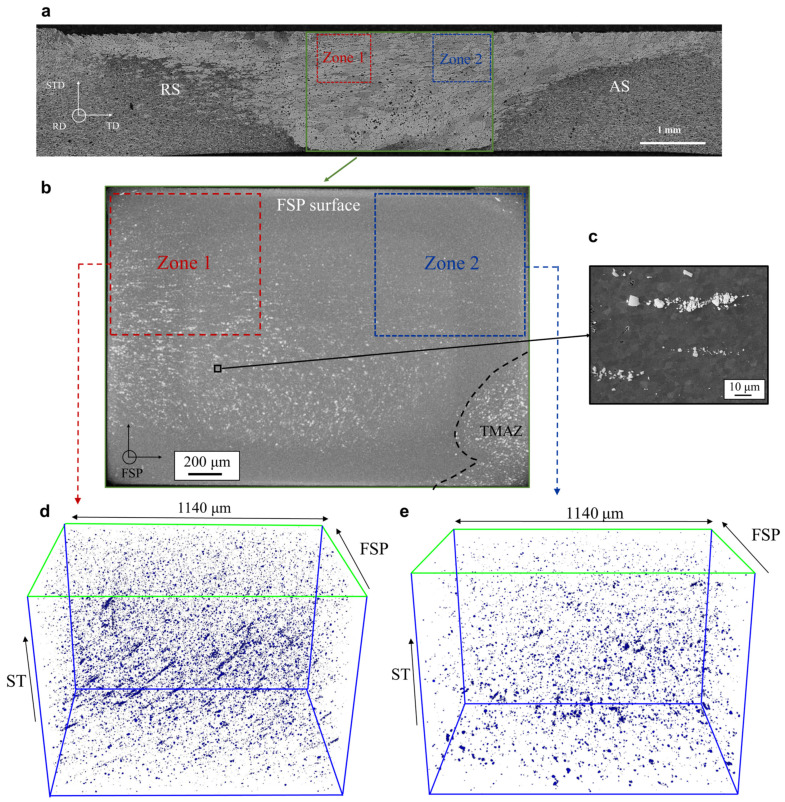
(**a**) Optical micrograph of the cross-section of the weld in a plane perpendicular to the FSP direction with visualization of the lower resolution XRCT specimen (green) and higher resolution XRCT sub-specimens (extracted from the FSPed stir zone in Zone 1 and Zone 2). (**b**) 2D visualizations of IM particles created by taking the maximum intensity projection (MIP) of the lower resolution (voxel size of 1.44 μm) XRCT images of the FSP-T6 sample over a 2 mm thick slab. (**c**) SEM micrograph with fragments of IM particles broken during FSP. 3D XRCT perspective of particles in the higher resolution XRCT image of the FSP sub-specimen in (**d**) the centre of the SZ and (**e**) the AS of the SZ. RD = rolling direction, TD = transverse direction, STD = short transverse direction.

Figure 3a and Figure 4b show 2D visualizations of IM particles within the matrix in BM and in FSPed, respectively. These 2D visualizations have been created by taking the Maximum Intensity Projection (MIP) of the lower resolution (voxel size of 1.44 μm) XRCT images of the samples over a 2 mm thick slab. In other words, all the IM particles in the XRCT volumes were projected along the rolling or FSP direction. Figure 3b and Figure 4b,c show similar 2D visualizations for the higher resolution (voxel size of 0.6 μm) XRCT images over a 0.8 mm thick slab.

Figure 3a,b shows that the distribution of IM particles within BM is quite homogeneous, but fewer particles are observed closer to the sample’s surfaces compared to the mid-thickness zone. Considering that the smallest particle, which can be reliably identified is around 4 μm (i.e., a diameter of 3 voxels), a lower particle content at the rolling surfaces could also be explained by smaller particles. This can be explained by previous observations on rolled Al sheets, i.e., IM particles are broken into smaller fragments closer to the rolling surfaces due to larger deformation compared to that of the core layer [28]. 

Figure 4a shows a cross-section of the FSP-T6 material, identifying the locations of the XRCT specimen and sub-specimens (Zone 1 and Zone 2). Comparing Figure 4b with Figure 3a, the surface fraction of IM particles appears to be lower in the FSP-T6 material. Since IM particles are broken into fragments during FSP [14,25], the lower particle content in the FSP-T6 projection is due to IM particle refinement and particle size becoming too small to be captured with a voxel size of 1.44 μm. This is also in good agreement with the particularly low IM particle content closer to the FSP surface, where particles are broken into even smaller fragments due to the large friction and pressure applied by the tool [13]. 

Figure 3c and Figure 4d,e show the reconstructed XRCT volume (observed with 0.6 μm voxel size) of BM as well as the retreating side and advancing side in the FSPed material, respectively. Particles and initial porosities are respectively highlighted in blue and red, as shown in Figure 3c. While a few porosities are observed in BM, none of these porosities are observed in the FSPed material at the resolution of the XRCT images. The volume fraction of initial porosity in BM is 0.0085%. The IM volume fraction (*F_v_*) is 0.23% and 0.05% in BM and in the FSPed material, respectively. Since these very stable IM particles are broken, but not dissolved during FSP [14], it means that approximately 80% of the IM particles’ volume fraction have been broken into fragments and were too small to be seen with 0.6 μm voxel size XRCT images. 

**Figure 5:**
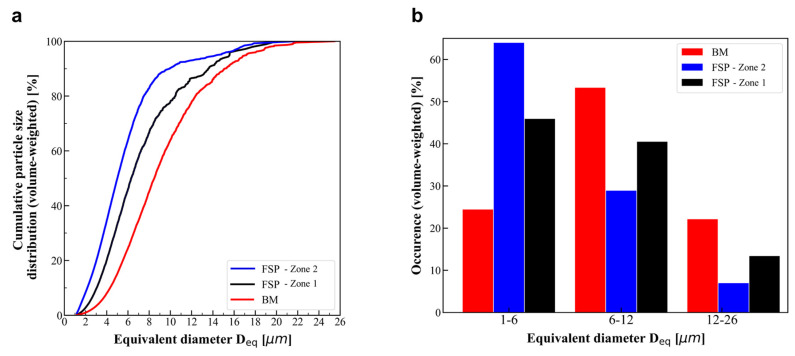
Intermetallic particle size distribution (volume-weighted) based on XRCT images. (**a**) Cumulative particle size distribution and (**b**) corresponding averaged parameters when dividing the IM size distributions into three size categories.

Figure 5a presents the cumulative size distributions for the IM particles based on 3D XRCT images (voxel size of 0.6 μm). The equivalent diameter *D_eq_* corresponds to the diameter of a sphere with the same volume as the particle. The size distributions are shifted to the left after FSP, indicating that IM particles are broken into smaller fragments. Figure 5b shows the corresponding averaged parameters when dividing the IM size distributions into three size categories. FSP reduces significantly the proportion of particles larger than 12 μm (Figure 5b), which are the most likely to fracture and nucleate voids during plastic deformation [4]. 

While large particles are efficiently broken into smaller fragments within the whole FSPed zone, it appears that the particle refinement and the redistribution of these fragments is not homogeneous. Figure 4 also shows that the particle size distribution is somewhat heterogenous within the FSPed zone. The particles found on the advancing side of the FSPed zone are broken into smaller fragments when compared to the retreating side. Furthermore, while IM particles are homogeneously distributed within the advancing side (see Figure 4d), particles are distributed into “lines” with a preferential orientation on the retreating side (see Figure 4e). These “lines” correspond to clusters of particle fragments, see Figure 4c showing a micrograph obtained by FEG-SEM within the FSPed zone. Their preferential orientation is probably due to the complex nature of the SZ material flow and the high shear strain within the FSP zones [12,13]. 

### 3.2. Mechanical Properties

#### 3.2.1. Hardness Profiles 

**Figure 6:**
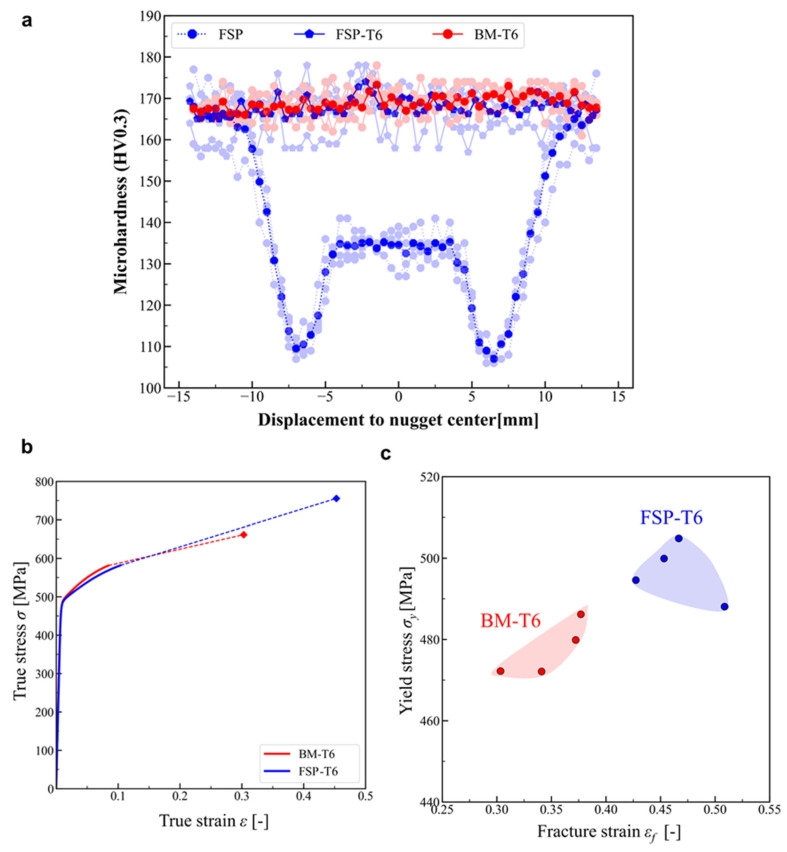
(**a**) Hardness measurements along the mid-thickness transverse direction in the BM-T6, as-FSPed and FSP-T6 materials. (**b**) Representative tensile true stress-strain curves obtained by loading BM-T6 and FSP-T6 materials and (**c**) results for all samples in terms of yield strength and fracture strain.

Figure 6a shows the hardness profiles along the mid-thickness transverse direction in the BM-T6, as-FSPed material and FSP-T6 material (i.e., after the post-processing heat treatment). The averaged hardness of the BM is around 170 HV0.3. The as-FSPed material is significantly softer than BM, with the mid-thickness hardness profile exhibiting an intermediate plateau (about 135 HV0.3) within SZ and two lowest peaks (about 110 HV0.3) close to the two TMAZs-HAZs transition zones. The main limitation of FSP to refine the microstructure of 7xxx Al alloys is related to this softening when processed in the T6 state. This phenomenon arises due to the dissolution of semi-coherent precipitates and the coarsening of incoherent precipitates upon exposure to the significant heat generated by friction and deformation [19]. Precipitates are dissolved in the matrix before dynamic recrystallization. During subsequent cooling, coarse precipitates nucleate preferentially on grain boundaries, as already observed in Figure 2d, and this precipitation coarsening induces a softening of the material [19]. 

After post-FSP T6 treatment, a homogeneous fine precipitation state is restored throughout the entire processed zone (see Figure 2f), and the hardness of the FSP-T6 material is very similar to the averaged value of the BM. Note that despite quite different grain structures (i.e., elongated and rather equiaxed in BM and FSP-T6, respectively), the hardness of the BM-T6 and FSP-T6 samples are very similar. This is due to the fact that the contribution of the grain size to the strengthening is negligible compared with the effect of precipitates in age-hardenable Al alloys.

#### 3.2.2. Uniaxial Tensile Test

Figure 6b shows a representative tensile true stress-strain curve obtained by loading BM-T6 and FSP-T6 materials. Figure 6c shows all samples in terms of yield strength and fracture strain. As shown in Figure 6c, all samples have similar yield stress around 480 MPa and 490 MPa, for BM-T6 and FSP-T6, respectively. This is in good agreement with similar hardness profiles observed for BM-T6 and FSP-T6 in Figure 6a. Figure 6b,c shows that the fracture strain of FSPed-T6 samples is significantly improved compared to BM-T6. The mean fracture strains are 0.34 and 0.47 for BM-T6 and FSP-T6, respectively, i.e., 38% improvement in fracture strain.

**Figure 7:**
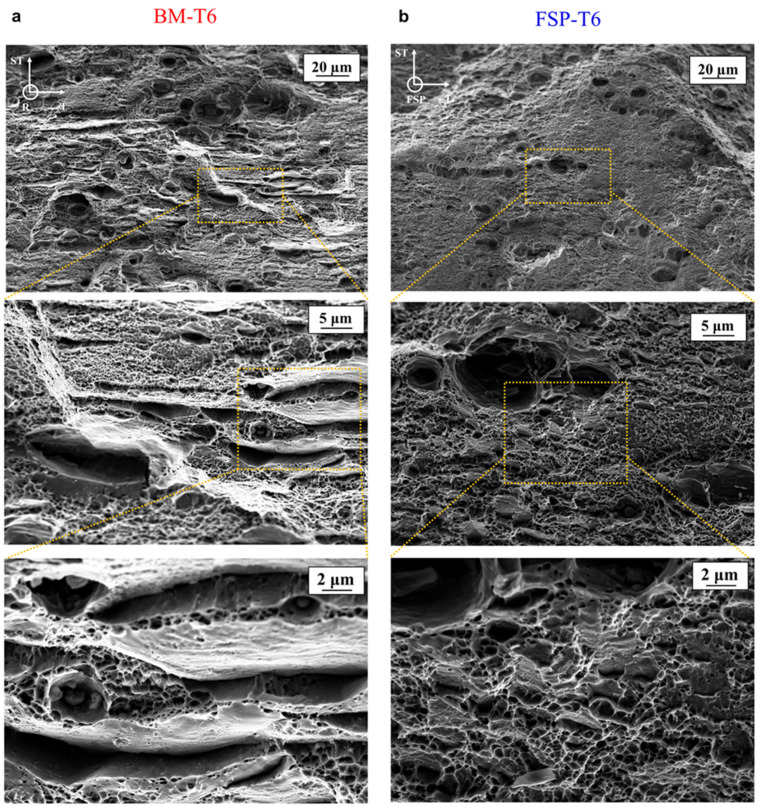
Fractography of uniaxial tensile specimens in the (**a**) BM-T6 condition and (**b**) FSP-T6 condition.

Figure 7 shows SEM micrographs of fracture surfaces for BM-T6 and FSP-T6 broken tensile specimens. In all specimens, many dimples are observed in the centre of the fracture surface, typical of ductile fracture. 

Figure 7a shows the fracture surface of the BM-T6. Broken IM particles are found inside coarse dimples. A population of finer dimples is also observed, associated with void nucleation on coarse precipitates. Furthermore, the grain decohesion mechanism is also active, as evidenced by flat and dimple-free areas on the fracture surface. Some coarse precipitates are visible on these detached grains, which are in good agreement with the coarse precipitation favoured along the grain boundaries (as discussed in Section 3.1.1). In other words, there is a competition between intergranular and transgranular failure in the BM-T6, and this is in good agreement with previous studies on 7xxx Al alloys [3,6]. 

Figure 7b shows the fracture surface of FSP-T6 material. The fracture surface is fully covered by larger and finer dimples, which are probably associated with void nucleation on micron-scale IM particles and sub-micron coarse precipitates, correspondingly. As opposed to BM-T6, intergranular failure by grain decohesion is not observed. The suppression of the intergranular failure mechanism is in good agreement with the improved ductility, i.e., fracture strain, observed for the FSP-T6 samples. 

#### 3.2.3. Plate Bending Tests 

**Figure 8:**
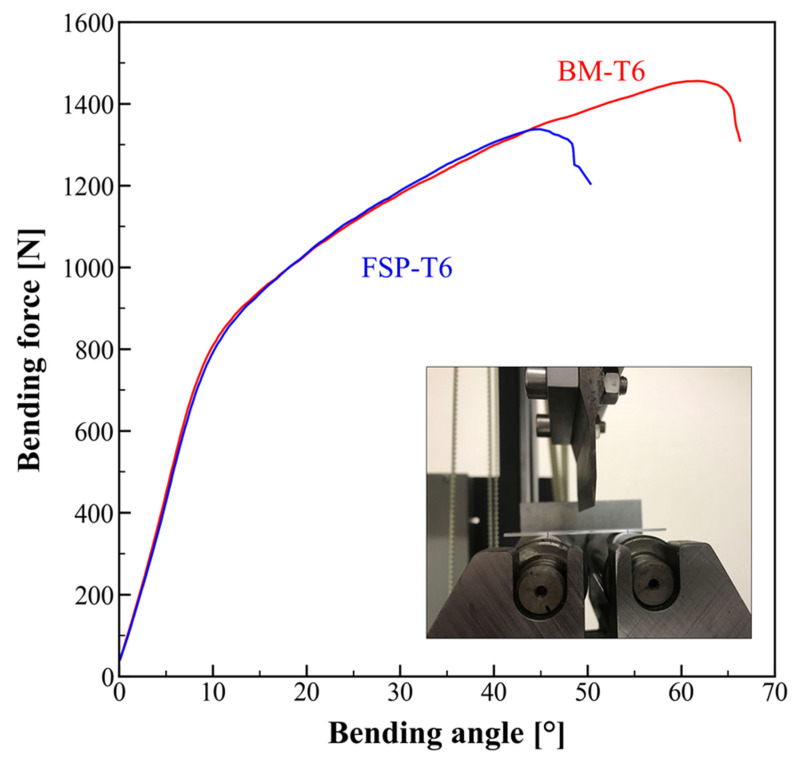
Representative bending curves with the punch force plotted as a function of the bending angle (following VDA-238-100-2017).

Figure 8 presents the representative bending curves with the punch force plotted as a function of the bending angle (following VDA-238-100-2017). Bending formability is usually quantified by the critical bending angle, which indicates that the metallic sheet cannot be bent through a larger bending angle than this critical value without cracking on the outer tensile surface. All FSP-T6 samples failed prior to BM-T6 samples, with a critical bending angle around 45° and 60°, respectively. 

**Figure 9:**
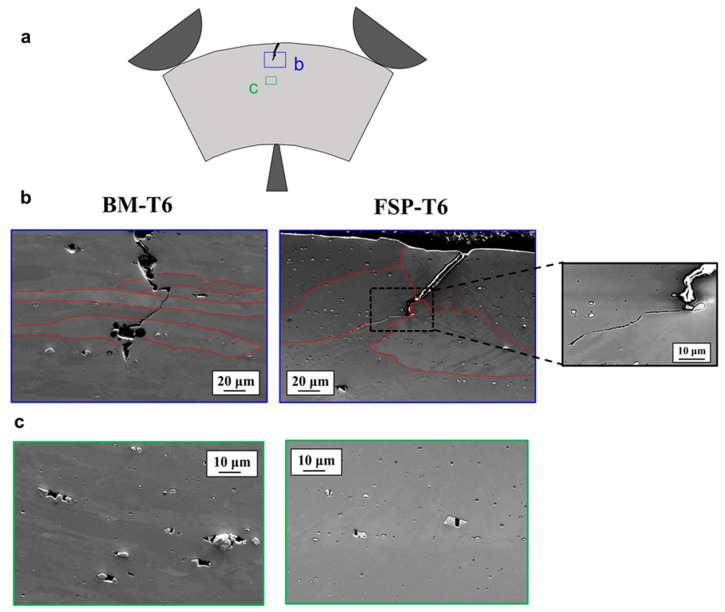
Bending coupons polished at mid-width after reaching the shut-off criterion (a 10% drop in force after the maximum force has been reached) in the BM-T6 and FSP-T6 bending specimens. (**a**) Schematic of the bending coupon with observation sites, (**b**) crack tip region, with grain boundaries highlighted in red, (**c**) regions in front of the crack tip, presenting broken IM particles and porosities.

Figure 9a shows a schematic of crack propagation during the bending test. Figure 9b shows SEM micrographs in front of the propagating crack tips, while Figure 9c shows SEM micrographs further away from the crack tip. Similarly, with what was observed on the tensile fracture surface of the BM-T6 sample, a competition between transgranular and intergranular failure modes is observed, see Figure 9b. In contrast, while the tensile fracture surface of the FSP-T6 was fully transgranular, it appears that the intergranular failure mode is significantly activated during the bending of the FSP-T6 sample. 

## 4. Discussion

While the tensile ductility was improved by about 40% for FSP-T6 samples compared to BM-T6, the opposite trend was observed in terms of formability, quantified by a 25% decrease in the critical bending angle. 

This is in contradiction to what has been traditionally observed in the literature, i.e., the bending formability usually increases with increasing fracture strain [22,29,30]. Datsko and Yang [22] proposed an empirical relationship between bendability and the reduction area, which is equivalent to the fracture strain, measured from a tensile test. This relationship is based on the assumption that failure occurs when the true strain in the outer tensile surface of the bent sheet is equal to the true fracture strain of a tensile test specimen. Note that since bending occurs in plane strain, the plane strain fracture strain should be used, but a similar relationship can be made with the tensile fracture strain [30]. Several experimental studies have shown a good agreement with this empirical relationship, such as in 6xxx Al alloys [29].

**Figure 10:**
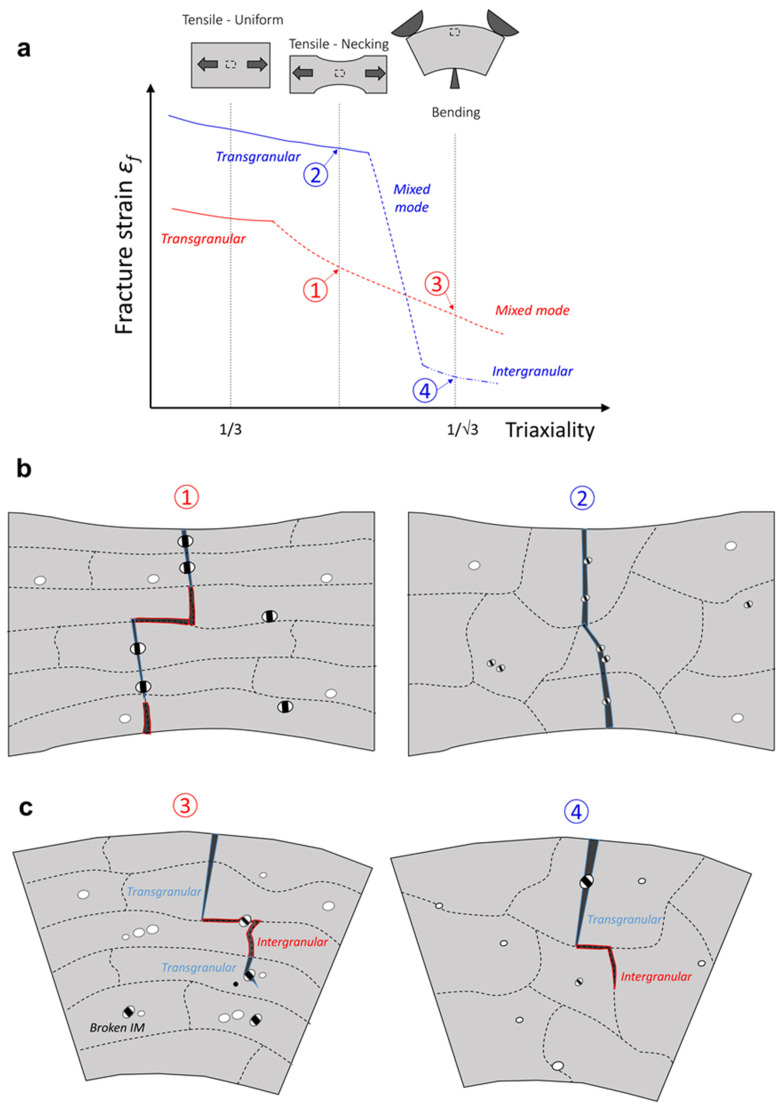
Schematic of the (**a**) effect of the global stress triaxiality (i.e., loading condition) on the failure mechanism in the BM-T6 (blue curve) and FSP-T6 material (red curve). Lower triaxiality points are representative of (**b**) uniaxial tensile (UT) specimens (T = 1/3). Higher triaxiality is representative of (**c**) plane strain bending specimen (T ~0.57).

The opposite trend observed in this study can be explained by the competition between intergranular and transgranular failure modes, depending on the material and on the stress state as schematically represented in Figure 10. 

The competition between intergranular and transgranular failure depends on the local stress state in PFZ and in the core of the grain. PFZs are softer and deform plastically prior to the interior of the grains. The elastic grains impose a strong constraint on these PFZs, which undergo a rapid and significant increase from the global stress triaxiality up to much larger local values (*T* = 2–3) after yielding [8]. In some cases, the grain starts to yield before the local failure of PFZ, the plastic flow inside PFZ becomes less constrained and the local stress triaxiality then decreases again. At this stage, there is a competition between the failure in the PFZ or in the grain, i.e., between intergranular and transgranular failure modes.

If PFZs undergo a much higher work hardening than the interior of the grain, then the PFZ induces a constraint on the plastic flow inside the grain, i.e., the local triaxiality within the grain increases, and the voids then tend to grow more rapidly. In this case, a damage-induced softening is then rapidly activated within the grain followed by void coalescence, i.e., transgranular failure mode is activated [9]. On the contrary, the work hardening of the material inside PFZs might not be sufficiently high compared with the interior of the grain [8]. A higher rate of void growth and associated void softening occurs within the PFZs. In this case, strain localization and formation of a crack take place along the PFZs, i.e., intergranular failure mode is activated and the transgranular mode is never observed. 

In a previous study [25], it was shown that FSPed-T6 Al 7475 presents PFZs with thickness around 25 nm, similar to PFZs observed in BM. Although PFZ imaging was not performed in this study, a similar trend is expected. This is also in good agreement with the fact that the strain hardening rates of the FSP-T6 and BM-T6 uniaxial tension (UT) specimens are not significantly different (see Figure 6b). Since PFZs have similar thicknesses, a difference in the work-hardening rate of PFZs cannot explain the different behavior between the BM and the FSPed materials.

The orientation of an anisotropic grain structure also influences the competition between failure mechanisms because the damage is favoured within PFZs when the major stress acts perpendicularly to the soft zones [8]. Kawabata and Izumi [31] also observed that intergranular fracture preferentially occurred on GBs, which are inclined at about 45° to the loading direction.

It is interesting to note that in an analysis based on finite element modelling of hexagonal-shaped grains, Fourmeau et al. [8] found that when loading the microstructure along the PFZs direction, there was a very limited influence of global stress triaxiality on the equivalent strain to failure for a triaxiality ranging between 0.3 and 0.6. In contrast, for the rotated microstructure with loading perpendicular to PFZs, the fracture locus exhibits a U-shaped dependence with a minimum ductility around a triaxiality of 0.6. 

This is in good agreement with our experimental observations (Figure 10). The BM microstructure is highly anisotropic, and the tensile stresses are mainly acting along the PFZs direction (both for uniaxial and bending specimens). Due to large IM particles in BM-T6 material, void nucleation is taking place at lower tensile strain when compared to the FSP-T6 [14,25]. Furthermore, there is a competition between intergranular and transgranular failure in the BM-T6 uniaxial tensile (UT) specimens. The fracture strain of the BM-T6 specimens is thus lower at low triaxiality (*T* = 1/3) than of the FSP-T6 UT specimens. 

During the bending of the thin sheets, the triaxiality is large (*T* ~0.57 [32]), but the effect of stress triaxiality on the failure mechanism is rather limited for BM. This is in good agreement with the results of Fourmeau et al. [8] where tensile stresses are mainly acting along PFZs. On the contrary, the fracture strain of the FSP-T6 UT specimen is significantly improved at low triaxiality (i.e., UT specimens), mainly due to a postponed void nucleation. However, the grain structure of FSP-T6 is rather isotropic, i.e., a large part of GBs and PFZs are perpendicular to the tensile stresses and the intergranular mode is activated. The increase of stress triaxiality induces an activation of the intergranular failure mode and there is a significant decrease of the equivalent strain to failure for bending samples, i.e., a decrease of the critical bending angle. 

Furthermore, the favoured intergranular mode for FSP-T6 at higher triaxiality (see Figure 10c) is also in good agreement with the results of Kawabata and Izumi [31]. They observed that intergranular fracture preferentially occurred on the GBs, which are inclined at about 45° to the loading direction. At low global stress triaxiality, the transgranular failure mechanism is favoured because it allows the increase of the stress in the grain’s core without too much void growth and void softening within PFZs [9]. 

Pardoen et al. [9] have shown with FE simulations that increasing the relative PFZ thickness compared to grain size has a marginal effect on the failure mode activated for a given global stress triaxiality. However, the PFZ thickness over the grain size ratio has a first-order effect on the fracture strain for a given failure mode. If PFZ thickness over grain size is decreased, the ductility is significantly improved at low triaxiality, i.e., for transgranular fracture mode. However, an increase in this ratio has the opposite effect at larger triaxiality, i.e., decreasing the ductility when intergranular failure prevails. Furthermore, the decrease of ductility observed at the transition from transgranular to intergranular is larger and sets in more abruptly for the smaller ratio of PFZ thickness over the grain size. Since PFZs are similar in BM-T6 and in FSP-T6, but with a larger grain size in FSP-T6, the PFZ thickness over the grain size is decreased in FSP-T6. The more abrupt decrease, larger fracture strain at low triaxiality and the lower fracture strain at large triaxiality for the FSP-T6 compared to BM-T6 (Figure 10) are thus well explained by the results of Pardoen et al. [9].

## 5. Conclusions

Friction stir processing and post-processing HT were performed on 7075 rolled Al sheets. The tensile tests revealed major improvement in the fracture strain for the same yield strength, while the bending formability was decreased. The main conclusions of this study are as follows:

1. Although the fragmentation of large IM particles occurs within the entire FSPed zone, the resultant particle refinement and redistribution are not uniform. From an applicability point of view, these results indicate that multi-pass FSP might be necessary to ensure a homogeneous microstructural refinement within a large volume.
2. The morphology and size of the grains were changed after FSP and post-processing T6 HT. The resultant FSPed-T6 material exhibited large equiaxed grains, measuring approximately 150 μm, which did not display any signs of abnormal grain growth.
3. Combining FSP and post-processing T6 HT results in significantly higher tensile ductility, as quantified by about a 40% increase of fracture strain in the FSP-T6 when compared to BM-T6. However, a systematic shift from transgranular failure mode in the case of uniaxial tensile specimens to intergranular failure in bending tests has been observed, limiting the formability of the FSPed-T6 material.
4. The competition between intergranular and transgranular has been explained by the effect of the global stress triaxiality depending on the microstructure. The stress triaxiality had a limited effect on the failure mechanism of the base material, showing a mixed intergranular-transgranular mechanism both in bending and under uniaxial tension. In contrast, the intergranular mode was favoured for FSP-T6 material at higher triaxiality while the transgranular failure mechanism was favoured at lower stress triaxiality under uniaxial tension.

## Figures and Tables

**Table 1:** Chemical composition of Al 7075.

Element	Zn	Mg	Cu	Mn	Ti	Cr	Zr	Si	Fe	Al
Wt.%	5.65	2.10	1.47	0.04	0.02	0.18	0.01	0.06	0.14	Bal.

## Data Availability

The tomography data can be found at https://github.com/H-S-T1/Tomography-data-for-Al7075 (accessed on 15 March 2023).
